# Could TNF-antagonists be a novel treatment strategy for BPH patients?

**DOI:** 10.15698/cst2022.06.268

**Published:** 2022-06-07

**Authors:** Renee E. Vickman, Omar E. Franco, Simon W. Hayward

**Affiliations:** 1 Department of Surgery, NorthShore University HealthSystem, 1001 University Place, Evanston, IL 60201 USA.

**Keywords:** benign prostatic hyperplasia, inflammation, autoimmune disease, tumor necrosis factor, therapy

## Abstract

Tumor necrosis factor (TNF) is widely recognized as a pivotal player in both systemic and local inflammatory processes. Due to the critical role this molecule has in driving both chronic and acute inflammation, it was among the earliest therapeutic targets utilized for patients with autoimmune (AI) diseases. While inflammation in the prostate is commonly observed, the organ has not previously been considered a target of systemic inflammation associated with some AI diseases. In patients with benign prostatic hyperplasia (BPH), chronic inflammation is common, and immune cells represent a significant proportion of cells in the organ. Accumulation of inflammatory cells may be a response to an initial insult and/or a factor in driving BPH pathogenesis. Certainly, inflammation can limit the efficacy of existing medical therapies in these patients. We previously showed that a pattern of gene expression in BPH tissues from patients who had progressed to indication-specific surgery was consistent with the changes seen in AI diseases. Recently, we demonstrated that patients with AI disease have an approximately 50% increase in BPH prevalence compared to patients without AI disease. Treatment of AI disease patients, specifically with TNF-antagonists, reduces BPH incidence back to, or in some diseases, below, the baseline population BPH diagnosis rate. Treatment of AI disease patients with the broad spectrum anti-inflammatory methotrexate did not elicit this reduction in diagnoses. Systemic treatment with TNF antagonists reduces epithelial proliferation and macrophage accumulation in the prostate tissues from two mouse models of prostatic hyperplasia as well as human patients. These studies suggest that TNF is a potential therapeutic target in BPH patients.

BPH becomes more prevalent in men with increasing age and is associated with prostatic enlargement and/or lower urinary tract symptoms (LUTS). Treatment strategies include either 5α-reductase inhibitors (5ARIs), which limit androgen receptor activity and cause the prostate to shrink due to increased apoptosis, or α-adrenergic receptor antagonists (α-blockers), which reduce smooth muscle tone and improve urine flow. Both treatment strategies can reduce LUTS in patients, thereby treating the clinical manifestation of BPH. Unfortunately, roughly one-third of BPH patients have no improvement in LUTS with therapy and some patients have recurring symptoms after a short-term improvement with treatment. There is a limited understanding of the mechanisms associated with BPH treatment failure, but chronic inflammation in the prostate has been associated with reduced therapeutic success in the Medical Therapy Of Prostatic Symptoms (MTOPS) and the REduction by DUtasteride of prostate Cancer Events (REDUCE) cohorts. In light of our recent studies, targeting inflammatory pathways with agents such as TNF-antagonists may provide an alternative or complementary therapeutic strategy for BPH/LUTS patients (**Figure 1**).

**FIGURE 1:**
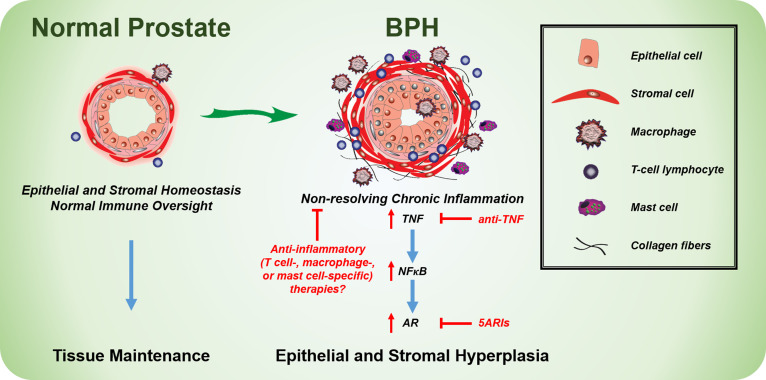
FIGURE 1: Model indicating the cellular changes in BPH compared to the normal prostate. Chronic inflammation is present in the prostates of many BPH patients, where anti-inflammatory therapies, such as TNF-antagonists, may be beneficial.

Even though our data show that TNF-antagonist treatment can reduce BPH diagnosis, treatment with methotrexate, commonly prescribed as a non-specific anti-inflammatory agent in a number of AI diseases, actually increases BPH incidence in AI disease patients. This finding is consistent with the limited responses previously noted with non-steroidal anti-inflammatory drug (NSAID) use in BPH/LUTS patients, suggesting a need for a deeper understanding of both the cellular and molecular connection(s) between prostatic inflammation and BPH.

The complete mechanism of TNF-mediated promotion of prostatic hyperplasia is unknown. Several driving factors for BPH development have been proposed as men age, including reduced testosterone:estrogen (T:E2) ratio, tissue remodeling, and loss of epithelial barrier integrity. Additional driving factors may lead to BPH and chronic inflammation concurrently, such as a response to damage-associated molecular patterns (DAMPs) or the development of an autoimmune response in the prostate (Figure 2). Anti-inflammatory interventions could be a useful therapy in combination with drugs targeting other aspects of this disease.

**FIGURE 2:**
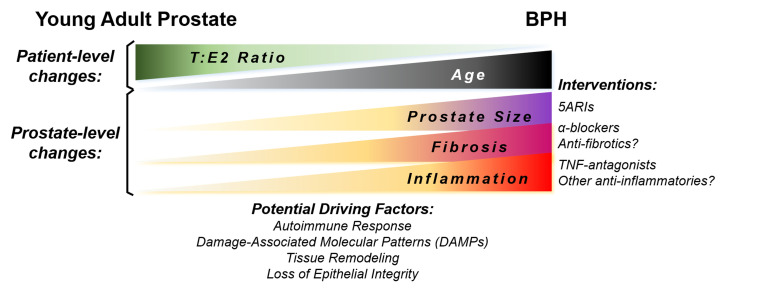
FIGURE 2: Long-term changes associated with BPH in men. This figure highlights classic changes in men during their lifetime, particularly those associated with the development of BPH. Potential drivers of prostatic changes and the relevant therapeutic interventions are listed.

After TNF-antagonist treatment in NOD mice, bulk RNA-seq of prostate tissues identified a reduction in inflammatory and anti-apoptotic pathways in treated versus control samples. TNF-antagonist treatment results in diminished inflammatory signaling. This was evident by a reduction in antigen presentation and differentiation of various T cell subsets, determined by KEGG pathway enrichment analysis. Similarly, TNF-antagonist treatment reduces signaling downstream of TNF receptor 2 (TNFR2), with a reduction in anti-apoptotic signals such as Jun1, IKK, and Akt. In a chronic inflammatory environment, activation of these factors may contribute to the enhanced proliferation observed in BPH. Thus, inhibition of these proteins through blockade of upstream factors such as TNF can restrict cell proliferation and overall prostatic growth and/or de-repress apoptosis and allow shrinkage of the gland. Multiple cell types can express TNF receptors, but scRNA-seq of all human prostatic cell types indicates the highest expression of TNFR2 in macrophages. Further investigations will determine if protein expression of TNFR2 matches the expression observed in the scRNA-seq data.

Our recent studies indicate that TNF can directly stimulate stromal, but not epithelial, proliferation *in vitro*, whereas TNF-antagonists reduce epithelial proliferation *in vivo*. It is likely that TNF derived from inflammatory cells (e.g. T cells, mast cells, or macrophages) functions either indirectly or in combination with other secreted factors to drive epithelial cell proliferation (**Figure 1**). Further work is necessary to elucidate mechanisms of inflammation-mediated regulation of prostatic hyperplasia.

An important aspect of patient management is recognizing the potential for co-morbidities in patients, in particular those generally seen by different medical specialists. In this regard, AI disease patients who have an elevated risk of developing BPH should perhaps be screened for urinary symptoms. Patients with psoriasis, rheumatoid arthritis, and type I diabetes are significantly more likely to be diagnosed with BPH after treatment with methotrexate versus no medication. On the other hand, psoriasis patients treated with TNF-antagonists have a significant reduction in BPH diagnosis rate compared to patients without AI disease. Together, these data indicate that specific AI disease therapies could either increase or decrease the likelihood of BPH diagnosis, even within the same disease. Of course, diagnosis of each AI disease may have a preferred therapeutic option that could differ from other AI diseases. The limited number of patients with some AI diseases in our recent studies provides an opportunity for a greater understanding of the connection with these diseases and BPH in larger patient cohorts. An additional evaluation of whether patients without AI disease but with other systemic inflammatory processes like obesity or type 2 diabetes could benefit from anti-inflammatory treatments requires further study. Our recent evaluation was conducted in a patient population that was primarily Caucasian, with only 4% African American representation. Considering possible health disparities across patient race/ethnicity, further work is needed to address potential differences.

It is not clear whether prostatic inflammation directly influences LUTS in BPH patients. Although many patients with very large prostates have moderate-severe LUTS, prostate size and symptoms do not directly correlate. Our recent studies did not include an evaluation of LUTS in animal models treated with or without TNF-antagonists (etanercept), so further studies are needed to evaluate if voiding function is improved after anti-inflammatory therapies. LUTS questionnaires developed by the American Urological Association (AUA) or the Lower Urinary Tract Dysfunction Research Network (LURN) provide beneficial clinical information, but are not able to provide insight into cellular changes within lower urinary tract tissues. Knowing if a BPH patient has chronic inflammation in the prostate could guide therapy. Identifying BPH patients who may benefit from anti-inflammatory therapies needs to be improved, perhaps through the use of imaging modalities or secreted biomarkers.

The hypothesis that BPH could be an autoimmune disease has been proposed previously. Our current studies do not specifically address this question, but do show a correlation between BPH and AI disease diagnoses. It is clear that inflammatory signaling plays a role in the hyperplastic response in the prostate, but more studies are required to determine whether inflammatory cell populations are autoreactive to prostate-specific proteins. BPH is both histologically and clinically variable among patients, so even if BPH is determined to have an AI disease phenotype, this may only be relevant to a subpopulation of patients. Indeed, in the medium to long-term, “BPH” is likely to become a term that describes a disease spectrum with variability in the hyperplastic cell type, symptoms, inflammatory milieu, presence of fibrosis, and prostatic enlargement (**Figure 2**). There is plenty of reason for optimism due to the advances in technology that allow for a cellular description of the disease within each patient, so in conjunction with expanding BPH biorepositories we have many opportunities to gain a greater understanding of this disease. Investigating whether TNF-antagonists have clinical benefit to a broad range of BPH patients is likely the first of many novel therapeutics to treat inflammation-associated BPH.

